# Temporal microbiome dynamics and fish health-associated dysbiosis in freshwater aquarium systems: a case study from River Wonders Singapore

**DOI:** 10.3389/fmicb.2026.1739391

**Published:** 2026-05-20

**Authors:** Xueyan Shen, Fendy Xiong Da Yu, Shangzhe Xie, Chia-Da Hsu, Jose A. Domingos, Susan Gibson-Kueh

**Affiliations:** 1 Tropical Futures Institute, James Cook University, Singapore, Singapore; 2 Centre for Sustainable Tropical Fisheries and Aquaculture, James Cook University, Townsville, QLD, Australia; 3 James Cook University, Singapore, Singapore; 4 Mandai Wildlife Group, Singapore, Singapore

**Keywords:** 16S rRNA sequencing, aquarium microbiome, fish health, maintenance practices, microbiome dysbiosis, opportunistic pathogens

## Abstract

**Introduction:**

Aquarium systems are engineered yet biologically dynamic ecosystems where microbial communities underpin nutrient cycling, organic matter decomposition and water quality regulation. These processes directly affect aquatic animal health.

**Methods:**

This study conducted a two-month time series analysis of waterborne microbiomes across six freshwater exhibits at River Wonders, Singapore, using 16S rRNA gene sequencing. Exhibits were categorized as “Healthy” (LM, MJ, EE) or “Stressed” (P, MJR, RG) based on fish health, and as indoor or semi-indoor/outdoor by system design.

**Results and discussion:**

Temporal fluctuations in microbial composition and diversity were evident over time, with distinct profiles between indoor and semi-indoor/outdoor. Potential opportunistic or pathogenic genera, including *Edwardsiella*, *Flavobacterium*, *Aeromonas*, *Pseudomonas* and *Mycobacterium*, were consistently among the 30 most abundant taxa. The most severe dysbiosis occurred in exhibit P, characterized by a transient *Pseudomonas* bloom (51.4%), loss of nitrifiers (*Nitrosomonas*, *Nitrospira*) and concurrent fish health issues. MJR and RG harbored persistent polymicrobial risks, while “Healthy” exhibits maintained relatively more balanced microbial communities with lower pathogen loads. Routine husbandry interventions (e.g., partial water changes, substrate cleaning) coincided with improved microbial evenness and reductions in opportunistic taxa. These findings highlight diagnostic potential of microbiome profiling to detect early dysbiosis and support evidence-based husbandry in managed aquatic systems.

## 1 Introduction

Microbial communities are essential to aquatic ecosystem function, mediating nutrient cycling, organic matter decomposition and water quality regulation (Krotman et al., 2020; Sehnal et al., 2021). In artificial systems such as public aquaria, maintaining microbial stability is critical, as dysbiosis can disrupt ecological processes, favor opportunistic taxa and compromise aquatic animal health (Van Bonn et al., 2015; Yajima et al., 2023). However, aquarium microbiomes are highly susceptible to disturbance from fluctuating bioloads, husbandry interventions, variable feeding regimes and new species introductions. These perturbations can destabilize microbial homeostasis, facilitate toxic metabolite accumulation (e.g., ammonia) and trigger pathogen proliferation, thereby increasing the risk of disease outbreaks and compromising animal welfare (Smith et al., 2012).

Ornamental fish in aquaria are vulnerable to diverse microbial infections (Chatterjee et al., 2020; Larcombe et al., 2025), with bacteria being the common cause of morbidity and mortality (Austin and Austin, 2016). Genera frequently implicated include *Aeromonas*, *Edwardsiella*, *Pseudomonas*, *Shewanella*, *Mycobacterium*, *Streptococcus*, *Flavobacterium*, *Plesiomonas* and *Vibrio* (Lewbart, 2001; Musa et al., 2008). Many of these bacteria exploit immunocompromised hosts or thrive under suboptimal environmental conditions (Roberts et al., 2009; Smith et al., 2012). Given that fish are continuously exposed to the surrounding microbial community, their gut microbiota is strongly shaped by environmental microbes (Sullam et al., 2012; Wong and Rawls, 2012; Xiong et al., 2018), with downstream impacts on digestion, immunity and disease resistance (Talwar et al., 2018; Xiong et al., 2018). High similarity between water and gut microbial assemblages has been documented in aquaculture fish species (Giatsis et al., 2015; Wong et al., 2013). Therefore, monitoring water microbiomes offers a noninvasive approach for the early detection of dysbiosis and disease risk in aquatic systems (Moran, 2015; Schmidt et al., 2015). However, the challenge lies in distinguishing reliable microbial indicators of fish health from background variability, as aquatic microbiomes are temporally dynamic and context dependent (Venter et al., 2004; Wang et al., 2012; Yajima et al., 2023). Advances in 16S rRNA gene sequencing now allow more precise characterization of microbial diversity and temporal dynamics (Maheshwari et al., 2024; Michaud et al., 2009), enabling detection of community shifts linked to environmental or biological stressors or disease (Kim et al., 2021; Xue et al., 2017).

River Wonders (formerly River Safari) in Singapore, Asia’s first river-themed wildlife park, house over 11,000 animals across 260 species, including iconic freshwater species such as arapaima, giant freshwater stingray, piranhas, electric eels, and endangered Mekong giant catfish and Antillean manatees. Despite advanced infrastructure, recurring disease events have been observed in several exhibits, suggesting that existing management may inadequately address the underlying microbiological or environmental drivers of fish health. This study conducted a two-month time series survey of waterborne microbial communities in six freshwater exhibits at River Wonders. The objectives were to (1) characterize microbial composition and dynamics within and between exhibits; (2) identify signatures of dysbiosis associated with potential disease risk; and (3) evaluate the impact of routine husbandry practices on microbial structure. The findings aim to provide insights into microbiome-informed management strategies to improve fish health and ecological resilience in managed aquatic systems.

## 2 Materials and methods

### 2.1 Sample collection and DNA isolation

Six freshwater exhibits at the River Wonders, Singapore, were selected for water microbiome monitoring. Exhibits were operationally categorized based on integrated qualitative assessments, including historical disease events, routine veterinary observations and husbandry records of fish condition during the study period. Specifically, three exhibits—Lake Malawi (LM), Mekong Jewel (MJ), and Electric Eel (EE)—were classified as “Healthy”, with no notable clinical signs, recent disease outbreaks, or unusual mortality during the monitoring period. In contrast, Piranha (P), River Gem (RG) and Mekong Jewel Rainbowfish (MJR) were classified as “Stressed”, where fish were observed to exhibit signs of suboptimal condition, such as visible lesions and abnormal pigmentation. No standardized quantitative health metrics were available across all systems, and therefore this classification represents an operational, observation-based categorization rather than a formal epidemiological assessment. EE, P and MJR are indoor closed recirculating systems; MJ and LM are semi-indoor; RG is a fully outdoor system. Exhibit features and representative species are summarized in Table 1.

**Table 1:** Overview of the six selected exhibits at River Wonders, Singapore.

Exhibit	Representative species	Salinity	Environment	Heath condition
Mekong jewel (MJ)	Bala shark; Jullien’s golden carp; Asian arowana; Thai mahseer (total 12 species; 111 individuals)	Freshwater	Semi-indoor	Healthy
Lake Malawi (LM)	Spindle hap; featherfin squeaker; bumble bee cichlid; giraffe hap (total 8 species; 233 individuals)		Semi-indoor	Healthy
Electric eel (EE)	Electric eel; colombian tetra (total 2 species; 16 individuals)		Indoor	Healthy
Piranha (P)	Redbelly piranha (total 1 species; 3 individuals)		Indoor	Stressed
River gem (RG)	Chinese barb; oneline tetra; denison barb; red phantom tetra; lemon tetra (total 25 species; 471 individuals)		Outdoor	Stressed
Mekong jewel-rainbowfish (MJR)	Boeseman’s rainbowfish; Red rainbowfish (total 2 species; 31 individuals)		Indoor	Stressed

Weekly water samples were collected from each exhibit between 21 Sep and 16 Nov 2023 to monitor temporal microbial dynamics. Additional samples were taken immediately before and after routine maintenance (e.g., 5–20% water changes, sand substrate vacuuming, glass cleaning) to examine short-term community shifts associated with these interventions. The timing of maintenance events varied among exhibits but each exhibit underwent routine maintenance at least once per week according to standard husbandry schedules. At each sampling time point, 1 L of water was collected from three spatially distributed locations per exhibit via sterile bottles and transported on ice to the laboratory. Water quality parameters, including pH, temperature (Tm, °C), dissolved oxygen (DO, mg/L), ammonia (mg/L), nitrite (NO_2_^−^, mg/L) and nitrate (NO_3_^−^, mg/L), were recorded concurrently, with values remaining relatively stable across the study period (Supplementary Table S1).

Water was filtered through 0.2 μm sterile membrane filters (SterlitechTM) in a Class II biosafety cabinet (ESCO). Filters were stored at −80 °C until DNA extraction using the DNeasy PowerWater Kit (50) (QIAGEN, Germany) following the manufacturer’s protocol. DNA quality was verified by 2% agarose gel electrophoresis, and DNA concentration was measured using both Nanodrop spectrophotometer and Qubit 2.0 fluorometer (Invitrogen, Carlsbad, CA, United States). The extracted DNA was stored at −20 °C for downstream processing. No extraction blanks or reagent-only controls were included during DNA extraction and library preparation.

### 2.2 Library preparation, sequencing and bioinformatic analysis

The V3-V4 hypervariable regions of the bacterial 16S rRNA gene were amplified using primers: forward (5′-CCTACGGRRBGCASCAGKVRVGAAT-3′) and reverse (5′-GGACTACNVGGGTWTCTAATCC-3′) (Ren et al., 2019). Indexed adapters were incorporated during PCR amplification. Amplicons were purified using Agencourt AMPure XP magnetic beads (Beckman Coulter, United States), quantified via a Qubit 2.0 fluorometer and assessed for fragment size distribution with an Agilent 2100 Bioanalyzer (Agilent Technologies, Palo Alto, CA, United States). The final libraries were pooled and sequenced on an Illumina NovaSeq 6000 platform (2 × 250 bp paired-end), following the manufacturer’s protocols (Illumina, San Diego, CA, United States). Base calling and image processing were performed via NovaSeq Control Software (MCS).

Raw reads were processed using the Quantitative Insights into Microbial Ecology (QIIME) pipeline^1^ (Caporaso et al., 2010). Paired-end reads were quality filtered and merged using QIIME v1.9.1, Cutadapt v1.9.1 and VSEARCH v1.9.6, requiring a minimum overlap of 20 bp. Adapter sequences were removed, bases with Phred scores <Q20 were trimmed from both ends, reads containing ambiguous bases (N) were discarded, and only merged sequences with a minimum length of ≥200 bp were retained. Chimeric sequences were identified and removed using the UCHIME algorithm. Read retention and removal are summarized in Supplementary Table S2. Quality-filtered sequences were dereplicated, and singleton sequences were removed prior to Operational Taxonomic Units (OTUs) clustering. OTUs were clustered at 97% similarity via VSEARCH (1.9.6) against the Silva 138 reference database^2^. OTU-based clustering was used at project initiation; ASV-based approaches offer improved error correction and higher sequence resolution and are recommended for future studies. Taxonomic classification was performed via the RDP classifier (version 2.2, https://sourceforge.net/projects/rdp-classifer). Alpha diversity metrics (Chao1, Shannon) were calculated in QIIME. Community structure was visualized via nonmetric multidimensional scaling (NMDS) on the basis of Bray–Curtis dissimilarity. Community-level differences among exhibits were evaluated using PERMANOVA (Adonis) and ANOSIM implemented in the vegan package in R, with analyses performed at each sampling time point. Redundancy analysis (RDA) was conducted in R (v4.2.3) via the vegan package with 999 permutations to examine correlations between microbial genera and environmental variables (temperature, DO, pH). Temporal variation in selected genera was assessed using Kruskal–Wallis tests across sampling time points (*p* < 0.05).

## 3 Results

### 3.1 Temporal variability in microbial diversity

**Figure 1:**
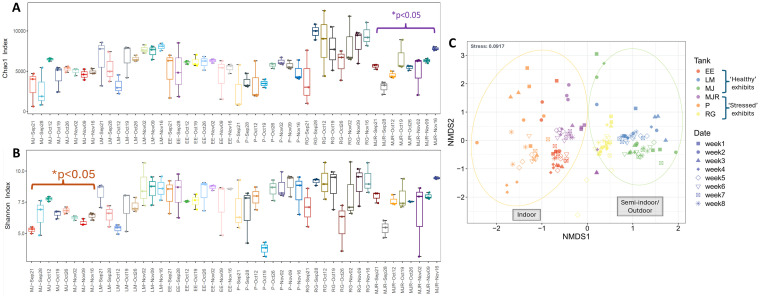
**(A)** Chao1 richness index over the two-month sampling period; **(B)** Shannon diversity index across sampling points, with statistically significant intra-exhibit differences denoted by an asterisk (*, *p* < 0.05). **(C)** NMDS ordination plot based on Bray–Curtis distances at the genus level, illustrating temporal clustering and inter-exhibit differences in microbial composition.

A total of 17,176,734 high-quality reads were generated from 186 water samples, averaging 92,348 reads per sample (Supplementary Table S2). Alpha diversity, assessed by OTU richness (clustered at 97% identity), Chao1 and Shannon indices exhibited pronounced temporal fluctuations both within and between exhibits. OTU counts ranged from 325 to 2,783 across all systems over time. Exhibits classified as “Stressed” (P and MJR) consistently shared fewer OTUs across time points, indicating reduced temporal stability relative to “Healthy” systems. Both Chao1 (Figure 1A) and Shannon (Figure 1B) indices showed intra-exhibit fluctuations over time, with statistically significant differences observed in Chao1 for MJR and in Shannon for MJ (*p* < 0.05; Figures 1A,B). Notably, a marked decline in Shannon diversity was observed in P on 19 October (Figure 1B). NMDS ordination revealed distinct clustering by exhibit (Figure 1C), with indoor systems (P, EE, MJR) separating from semi-indoor/outdoor systems (MJ, LM, RG). Among all exhibits, P displayed the most dispersed clustering pattern, consistent with high temporal variability in community composition (Figure 1C). PERMANOVA (Adonis) analysis confirmed significant differences in microbial community composition among exhibits (*p* ≤ 0.05) and ANOSIM analysis showed similar results, supporting significant separation among exhibits.

### 3.2 Bacterial phyla dynamics

**Figure 2:**
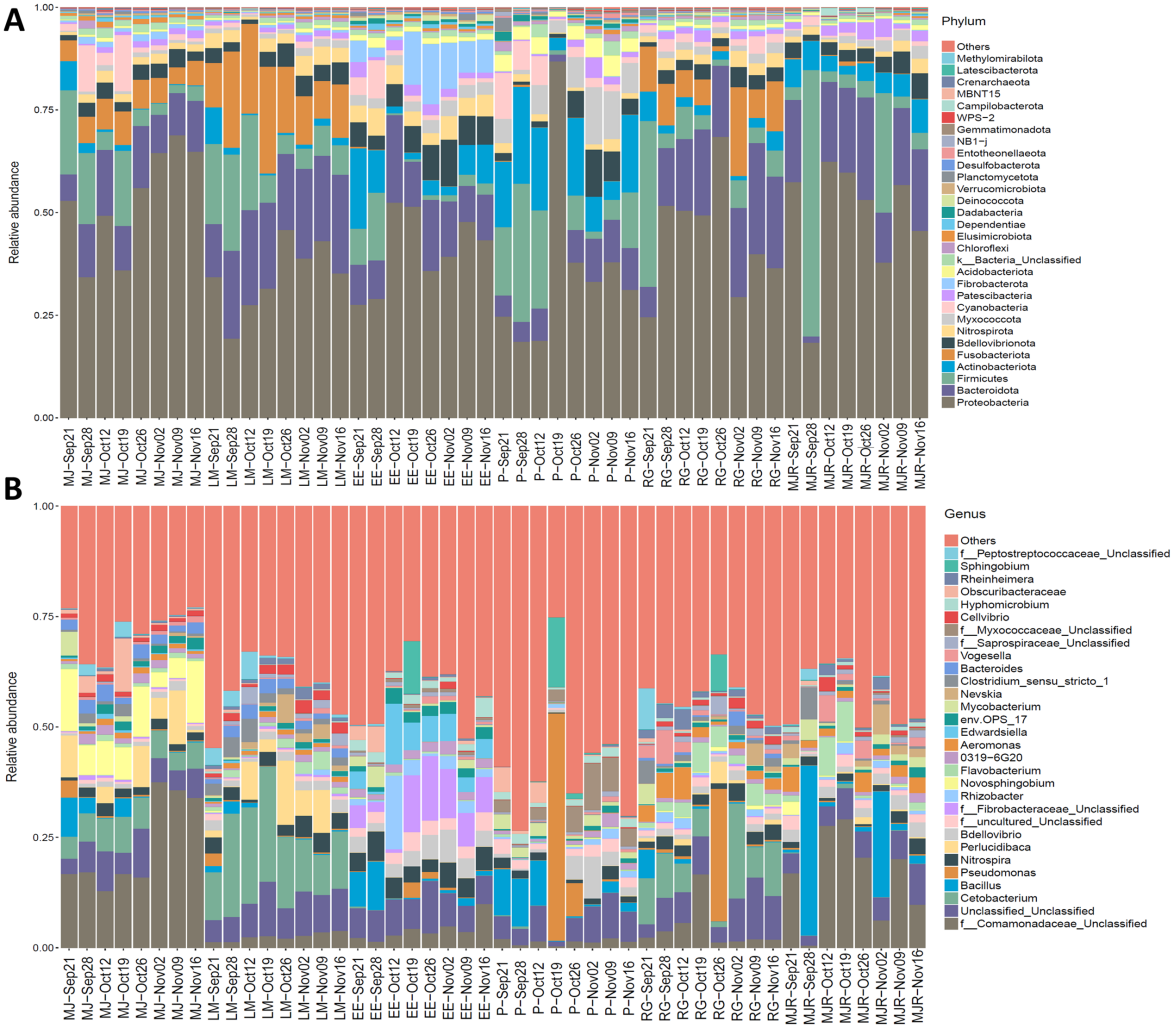
**(A)** Relative abundances of the 30 most abundant bacterial phyla across water samples from the six exhibits. **(B)** Relative abundances of the 30 most abundant genera across the same exhibits. The MJ, LM, EE, P, RG, and MJR host unique resident species.

Semi-indoor/outdoor exhibits (MJ, LM, RG) were consistently dominated by *Proteobacteria*, *Firmicutes*, *Bacteroidetes* and *Fusobacteriota*, with additional contributions from *Actinobacteriota*, *Bdellovibrionota*, *Nitrospirota*, and *Cyanobacteria*. These eight phyla collectively represented over 90% of the relative abundance in MJ, LM and RG (Figure 2A). Temporal variation in dominant phyla was observed across exhibits. In MJ, *Proteobacteria* varied from 34.3 to 68.8%, *Firmicutes* from 0.2 to 20.5%, and *Cyanobacteria* from 0.9 to 11.3%. LM exhibited temporal shifts in *Proteobacteria* (19.3–45.8%), *Firmicutes* (4.6–23.5%), *Fusobacteriota* (0.9–26.0%) and *Bacteroidetes* (4.6–24.5%). In RG, *Proteobacteria* fluctuated between 24.6 and 68.4%, *Firmicutes* between 1.5 and 40.4%, and *Fusobacteriota* between 1.2 and 11.0%. In contrast, indoor exhibits (EE, P, MJR) showed consistently lower or undetectable *Fusobacteriota* and relatively elevated proportions of *Actinobacteriota* (Figure 2A). EE was enriched in *Fibrobacterota* (up to14.6%), whereas P intermittently accumulated *Myxococcota* (up to 13.9%). Notably, P experienced a transient spike in *Proteobacteria* to 86.8% on 19 Oct, accompanied by declines in *Firmicutes*, *Bacteroidetes*, and *Actinobacteriota*. MJR showed a distinct *Firmicutes* peak (64.9%) on 28 Sep, concurrent with a marked decrease in *Bacteroidetes*.

### 3.3 Prevalence of opportunistic and pathogenic bacterial genera

**Figure 3:**
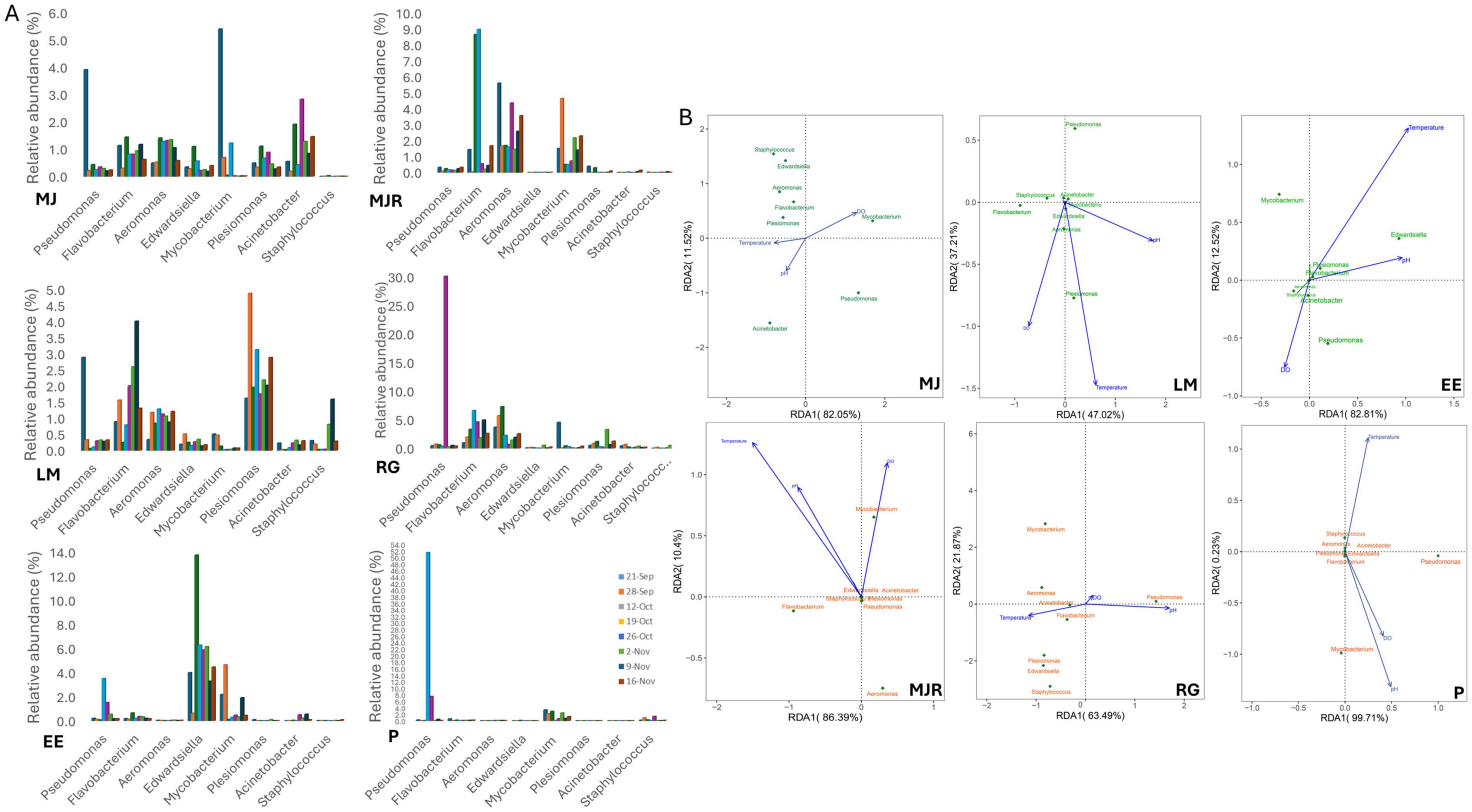
**(A)** Temporal dynamics of the eight genera across sampling points. **(B)** RDA plot illustrating genus-level associations with environmental parameters (temperature, DO, pH). Genus labels are colored in green and orange. Blue arrows represent environmental vectors; angles <90° indicate positive correlations, >90° indicate negative, and perpendicular vectors indicate no correlation.

Genus-level profiles revealed exhibit-specific microbial signatures, with relatively stable within-exhibit diversity but marked temporal shifts in the relative abundance of certain taxa (Figure 2B). Five genera previously reported in association with fish disease contexts, including *Edwardsiella*, *Flavobacterium*, *Aeromonas*, *Pseudomonas* and *Mycobacterium*, were consistently among the top 30 taxa and exhibited pronounced spatiotemporal variation, particularly in exhibits classified as “Stressed” (P, MJR, RG) (Figure 3A). Additional genera such as *Plesiomonas*, *Acinetobacter* and *Staphylococcus* were also detected at varying abundances.

“Stressed” systems displayed pronounced instability. In exhibit P, *Pseudomonas* spiked to 51.4% on 19 Oct and remained elevated (7.4%) on 26 Oct, while remaining below 0.4% at other time points (Figures 2B, 3A). Kruskal-Wallis tests confirmed significant temporal variation in *Pseudomonas* abundance within exhibit P (*p* < 0.05), with the highest abundance observed on 19 Oct. *Mycobacterium* was detected persistently at ~2.2 to 3.0% through mid-Oct and re-emerged on 2 Nov (2.4%). *Staphylococcus* peaked at 1.4% on 26 Oct., with other genera appearing sporadically at low levels. In MJR, *Aeromonas* remained consistently abundant (1.5–5.6%), while *Flavobacterium* peaked on 12 and 19 Oct (8.7 and 9.0%), with abundances significantly higher than at other sampling points (*p* < 0.05), yielding a combined relative abundance exceeding 11% on both dates (Figures 2B, 3A). *Flavobacterium columnare* alone accounted for 3.9% on 19 Oct. *Mycobacterium* reached 4.6% on 28 Sep., comprising *M. fortuitum* (2.0%) and an unclassified *Mycobacterium* sp. (1.4%). In RG, *Pseudomonas* surged to 30.1% on 26 Oct, significantly higher than the other sampling points (*p* < 0.05), coinciding with a rise in *Flavobacterium* (4.6%), together representing 34.7% of the community (Figures 2B, 3A). *Aeromonas* ranged from 1.4 to 7.3%, and *Plesiomonas* was consistently detected, with *P. shigelloides* peaking at 3.3% on 02 Nov.

In contrast, exhibits classified as “Healthy” generally harbored lower relative abundances of these genera. In LM, *Flavobacterium* reached 4.0% on 09 Nov, *Aeromonas* 1.3% on 19 Oct, and *Plesiomonas* ranged from 1.6 to 4.9% (with *P. shigelloides* at 4.7% on 28 Sep) (Figures 2B, 3A). In MJ, *Mycobacterium* (5.4%) and *Pseudomonas* (3.9%) peaked on 26 Oct, while other genera were detected at low abundance, except *Acinetobacter*, which reached 2.8% on the same date. In EE, *Edwardsiella* dominated most time points (3.3 to 13.7%), except for a transient decline to 0.6% on 28 Sep and a peak on 12 October (13.7%), which was significantly higher than at other time points. The highest combined abundance of the eight genera occurred on 12 Oct (14.7%), driven by primarily *Edwardsiella*. *Mycobacterium* peaked at 4.6% on 28 Sep and was also detected at lower levels on 21 Sep (2.2%) and 9 Nov (1.9%). Other pathogenic and opportunistic genera were sporadically detected at low abundance (Figure 2B, 3A). Beneficial nitrifying genera were also tracked. *Nitrospira* was detected in all exhibits, with highest abundances in LM (4.8% on 02 Nov) and EE (6.7% on 28 Sep). In MJ, *Nitrospira* initially dropped to 0.7% but subsequently stabilized between 1.5–2.9%. RG maintained relatively stable levels (1.2–3.9%), whereas P and MJR showed lower values. *Nitrosomonas* was detected sporadically at low abundance (<0.3%) across only RG and LM. To further illustrate exhibit-specific temporal variation, a heatmap of selected dominant genera was generated across all sampling time points (Supplementary Figure S2), highlighting changes in relative abundance patterns within each exhibit over the 8-week period.

Redundancy analysis (RDA) revealed exhibit-specific correlations between bacterial genera and environmental parameters (temperature, DO, pH) (Figure 3B). In P, *Pseudomonas* was positively associated with all three parameters, while *Mycobacterium* correlated positively with DO and pH but negatively with temperature. In MJR, *Mycobacterium* showed positive associations with all measured parameters, while *Aeromonas* displayed opposite trends, and *Flavobacterium*, which aligned with temperature and pH but opposed DO. In RG, *Pseudomonas* aligned with DO and pH, while *Mycobacterium*, *Edwardsiella*, *Plesiomonas* and *Staphylococcus* aligned with temperature but opposed pH. In EE, *Edwardsiella* was positively associated with both temperature and pH. In LM, most genera clustered near the origin, indicating weak associations with measured variables, whereas in MJ, taxa exhibited diffuse but broadly positive alignment with all variables.

### 3.4 Routine maintenance increased bacterial diversity and reduced abundance of opportunistic taxa

**Figure 4:**
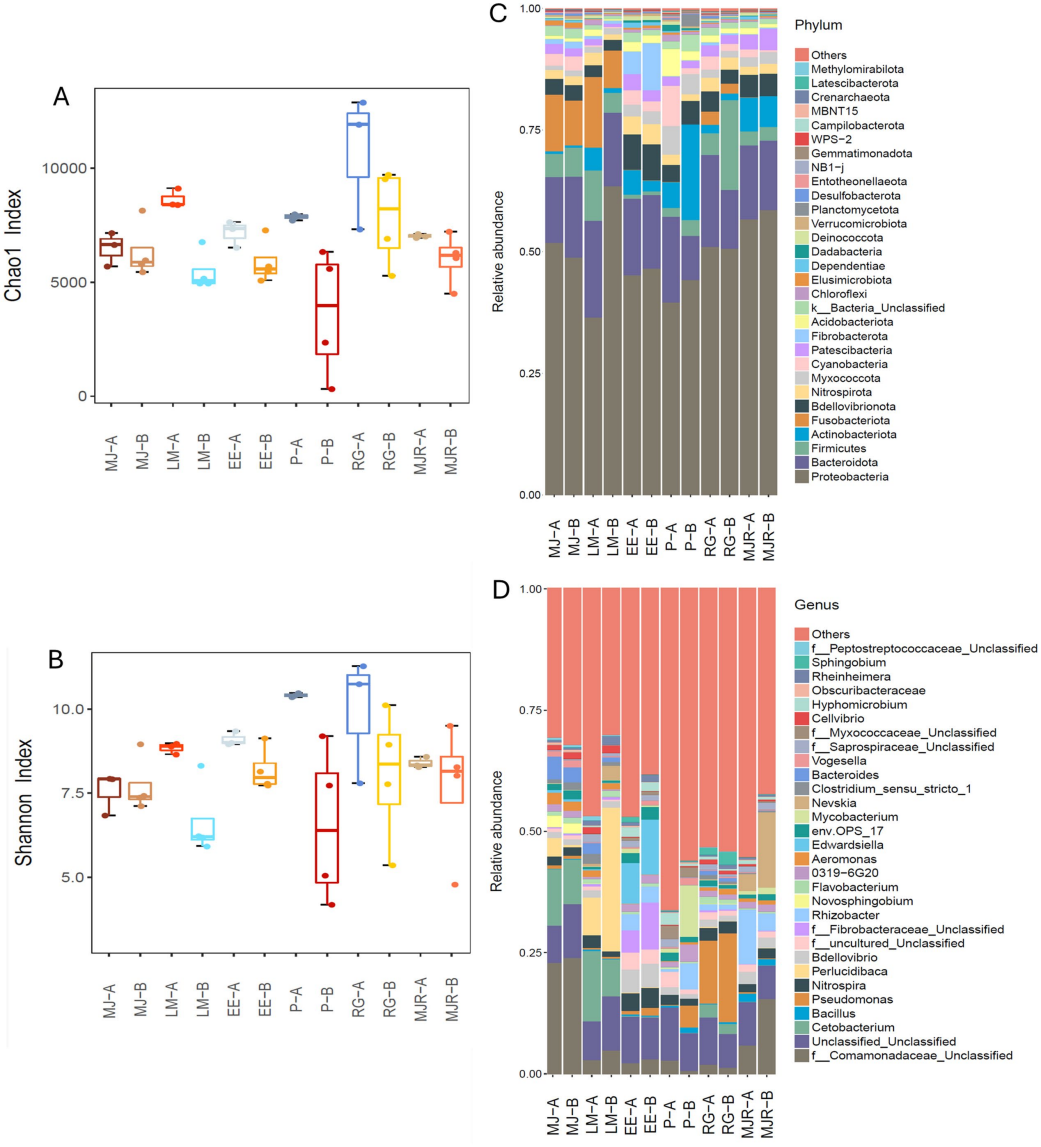
Impact of maintenance on bacterial community diversity and composition. **(A,B)** Chao1 and Shannon indices before and after maintenance. **(C,D)** Relative abundances of dominant bacterial phyla and genera before and after maintenance. Exhibits are labeled MJ, LM, EE, P, RG, and MJR, with “-B” (before) and “-A” (after) denoting timepoints relative to maintenance activities.

To assess the effects of routine husbandry maintenance, microbial alpha diversity and community composition were compared before and after interventions. Across multiple exhibits, maintenance was consistently associated with increased alpha diversity, with Chao1 richness increasing by approximately 1.0–2.2-fold and Shannon diversity increasing 1.0–1.6-fold relative to pre-maintenance levels (Figures 4A,B). These changes were accompanied by measurable shifts in overall community composition (Figures 4C,D). At the genus level, maintenance coincided with reduced relative abundance of several taxa commonly reported as opportunistic or conditionally pathogenic in aquatic systems. *Pseudomonas* declined from 1.6 to 0.7% in EE, 4.5 to 0.1% in P and 13.0 to 8.3% in RG after maintenance. Similarly, *Edwardsiella* decreased from 11.3 to 8.3% in EE, while *Mycobacterium* was reduced from 10.6 to 0.7% in P and from 1.3 to 0.7% in MJR post maintenance (Figure 4D).

## 4 Discussion

Microbial dysbiosis in aquatic environments is increasingly recognized as a driver associated with disease susceptibility in fish, with important implications for both public aquarium management and aquaculture production systems (Guo et al., 2022; Li et al., 2021; Xavier et al., 2024). In this two-month longitudinal survey of six freshwater exhibits at River Wonders Singapore, we characterized temporal shifts in waterborne microbial communities. Beyond documenting taxonomic variation within and between exhibits, the observed exhibit-specific trajectories provided insight into processes linked to ecological destabilization, the proliferation of potentially opportunistic and pathogenic taxa, and the influence of husbandry practices on microbial resilience in managed freshwater systems.

Exhibit P displayed the most pronounced microbial imbalance, characterized by a transient bloom of *Pseudomonas* (51.4%) coinciding with a collapse of the nitrifying taxa *Nitrosomonas* and *Nitrospira*. In aquatic systems, disruption of nitrification has been associated with ammonia accumulation, a known physiological stressor that can impair gill function and induce oxidative stress in fish (Ciji and Akhtar, 2020; Godzieba et al., 2024). Such shifts in nitrifying communities may contribute to microbial network destabilization and create conditions favorable for the proliferation of opportunistic facultative anaerobes such as *Pseudomonas*. Several species within the genus *Pseudomonas* are recognized opportunistic fish pathogens capable of causing systemic infections when host immunity is compromised by environmental or physiological stressors (Ardura et al., 2013; Duman et al., 2021). For example, *P. aeruginosa* has been reported to cause hemorrhagic septicemia, gill necrosis and severe systemic disease in teleosts (Ali et al., 2021; El-Gammal et al., 2025; Hussein et al., 2023). The potentially opportunistic taxon *Mycobacterium* was persistently detected in exhibit P, where tuberculosis-like pathology was intermittently observed in resident redbelly piranhas. Recurrent detection at the genus level is consistent with patterns reported in chronic mycobacterial infections in fish, including those associated with *M. marinum*, *M. chelonae* or *M. fortuitum* (Delghandi et al., 2020; Puk et al., 2018; Zanoni et al., 2008). Such infections are of particular concern due to their chronic nature and potential biosecurity implications for both fish and animal handlers (Gauthier and Rhodes, 2009; Hashish et al., 2018). It should be noted that 16S rRNA gene sequencing, particularly the OTU-based pipeline used in this study, primarily resolves taxa to the genus level (Bailén et al., 2020; Watts et al., 2017). Establishing causal links between specific microbial taxa and disease outcomes will require future studies integrating culture-based isolation, species- or strain-specific molecular diagnostics, and standardized health metrics.

Exhibit MJR displayed recurrent signatures of microbial imbalance, with repeated detection of potentially opportunistic taxa, including *Aeromonas* and *Mycobacterium*, and episodic increases in *Flavobacterium* (up to 9.0%). These genera include taxa that are frequently reported as primary or opportunistic fish pathogens (Pattanayak et al., 2020; Tomás, 2012), and their co-occurrence in this exhibit may reflect cumulative environmental stress and an elevated potential for polymicrobial instability. The detection of *Flavobacterium columnare,* a broad-host-range pathogen associated with columnaris disease (Declercq et al., 2013), is notable given its reported impacts across more than 37 freshwater species and its association with external lesions affecting skin and gill tissues (Ahmed et al., 2025; Hussein et al., 2023; Noga, 2010). Such clinical signs were intermittently observed in the exhibit, although direct causality cannot be established based on the present data. Similarly, exhibit RG demonstrated transient co-blooms of potentially opportunistic taxa, including *Flavobacterium* and *Aeromonas*, along with episodic increases in *Plesiomonas*, *Pseudomonas* and *Edwardsiella* (35.6% on 26 Oct). *Aeromonas hydrophila,* a common opportunistic pathogen, has been widely reported as a stress-associated taxon that can express increased virulence under immunocompromised conditions (Leung et al., 1995; Pattanayak et al., 2020; Snieszko, 1974; Tomás, 2012). In addition, *Plesiomonas shigelloides* (3.3% on 02 Nov), previously associated with disease in ornamental cichlid disease (Nisha et al., 2014), has been implicated in disease outbreaks across various fish species, both as a primary agent (Chen et al., 2022; Duman et al., 2023; Pękala-Safińska, 2018) and as part of polymicrobial infections involving *Flavobacterium* spp., *A. hydrophila* and *Edwardsiella* spp. (Fuentes-Valencia et al., 2022; Pękala-Safińska, 2018). Collectively, these temporal patterns suggested that increased disease risk in exhibits MJR and RG may be associated with episodic dominance of polymicrobial consortia under cumulative environmental stress, consistent with the higher microbial instability observed in exhibits classified as “Stressed.” Nevertheless, given the observational nature of the dataset and weekly sampling resolution, causal relationships between microbial dynamics and disease outcomes cannot be directly inferred.

By contrast, exhibits classified as “Healthy” generally tended to maintain more balanced microbial communities with lower relative abundance of opportunistic and pathogenic taxa, consistent with observed favorable fish health status. Nevertheless, the genus *Edwardsiella*, which includes known opportunistic pathogens, was persistently detected in exhibit EE, reaching relative abundances of up to 13.7%, suggesting a potential latent risk. *Edwardsiella tarda* is a well-documented fish pathogen associated with systemic infections and significant economic losses in aquaculture systems (Kerie et al., 2019; Miniero Davies et al., 2018), and it is also recognized as zoonotic agent (Michael and Abbott, 1993). These observations suggested the potential value of continued microbiome monitoring even in outwardly stable systems, as latent microbial risks may not be readily apparent from visual health assessments alone. In addition, the absence of extraction blanks and reagent (mock) controls represents a limitation, particularly in water microbiome studies where contamination may influence the detection of low-abundance taxa; therefore, such taxa should be interpreted with caution.

NMDS analysis showed a clear divergence between indoor (EE, P, MJR) and semi-indoor/outdoor (MJ, LM, RG) exhibits, indicating distinct bacterial community structures associated with exhibit type. Semi-indoor/outdoor exhibits were enriched in *Fusobacteriota*, dominated by *Cetobacterium*, *a* gut-associated genus involved in vitamin B12 synthesis and host metabolism (Qi et al., 2023; Tsuchiya et al., 2008; Wang et al., 2021). This enrichment pattern was consistent with higher host-derived organic inputs associated with larger and more diverse fish assemblages in these exhibits (Table 1), which may promote the accumulation of fecal-associated microbes (Bharti et al., 2023; Giatsis et al., 2015). However, given the observational nature of the study, other co-varying factors, including feeding regimes, biomass density and husbandry practices, may also contribute to this pattern and cannot be independently resolved. In contrast, indoor exhibits showed a relatively higher representation of *Actinobacteriota* (formerly *Actinobacteria*), a phylum that includes taxa adapted to oxygen-rich environments and noted for producing antimicrobial metabolites *(*Dmitrieva et al., 2023*).* This enrichment was consistent with the stronger aeration and lower organic loading typically associated with enclosed systems. In aquaculture, selected *Actinobacteria* strains, particularly *Streptomyces*, have demonstrated probiotic and bioaugmentation potential, including quorum-quenching against pathogens and enzymatic degradation of organic waste that supports water quality (Ghosh et al., 2023; James et al., 2023). However, *Actinobacteriota* also encompass pathogenic or opportunistic lineages, such as *Mycobacterium* (James et al., 2023; Seshadri et al., 2022). The recurrent detection of *Mycobacterium* in indoor systems, particularly exhibit P, may potentially reflect a compositional feature associated with enclosed aquatic environments with potential health relevance.

Routine husbandry practices, including partial water changes and substrate vacuuming, were associated with short-term increases in microbial richness and evenness across exhibits, together with reduced relative abundance of several genera previously linked to opportunistic behavior including *Pseudomonas* (P and RG), *Edwardsiella* (EE) and *Mycobacterium* (P and MJR). Physical cleaning may contribute to microbial rebalancing by reducing organic load and disrupting biofilms, thereby suppressing potential pathogen reservoirs, as suggested in other aquatic systems (Bik et al., 2019; Shade et al., 2012; Van Bonn et al., 2015). However, these observations were based on limited pre- and post-maintenance comparisons and replicated intervention studies would be required to robustly quantify the effects of husbandry practices. Environmental parameters remained broadly stable at weekly sampling resolution throughout the study. Accordingly, RDA associations likely reflected broad environmental gradients and exhibit-level structuring rather than direct drivers of acute dysbiosis events. Despite this, exhibit-specific microbial-environment associations were detected, consistent with reports from freshwater aquaculture ponds where seasonal variation structured microbial communities (Debnath et al., 2025), *Scylla paramamosain* culture systems with strong microbial-environment interactions (Zhou et al., 2025), and gut microbiota of salmons (Neuman et al., 2016; Zhao et al., 2021) and rainbow trout (*Oncorhynchus mykiss*) (Huyben et al., 2018), where water quality influenced microbial community organization. From a community assembly perspective, microbial structure is shaped by the combined effects of diversification, dispersal, selection, drift and historical interactions (Ho et al., 2016; Nemergut et al., 2013). The clustering of multiple genera near the RDA origin in this study suggested that abiotic variables alone may not fully explain waterborne microbiome trajectories, and that unmeasured biotic interactions or system-specific factors may also contribute. In this context, longitudinal microbiome profiling may provide a complementary indicator of system stability in managed aquatic environments. Higher frequency environmental monitoring and replicated husbandry interventions would be required to resolve fine-scale microbial-environment dynamics and test causal relationships.

## 5 Conclusion

This study provided the first longitudinal characterization of waterborne microbiomes in freshwater exhibits at River Wonders, Singapore. Exhibits classified as “Stressed” showed greater microbial instability and enrichment of opportunistic taxa, whereas “Healthy” exhibits maintained more balanced community structures. Routine husbandry practices were temporally associated with increased microbial evenness and reduced relative abundances of several opportunistic genera, highlighting their potential role in supporting system stability. Although genus-level taxonomic resolution of the dataset, these findings demonstrated the value of waterborne microbiome profiling as a non-invasive indicator of ecological imbalance in managed aquatic systems. Future studies integrating host-associated microbiomes, functional metagenomics, standardized health metrics and long-term monitoring will be required to establish causal relationships and identify microbial drivers of health and resilience. Overall, the observed patterns aligned with findings from aquaculture ponds and recirculating systems, underscoring the broader relevance of waterborne microbiome monitoring for proactive aquatic health management.

## Data Availability

The sequences generated of 16S rRNA genes are available via the NCBI public repository under the BioProject accession PRJNA1290883 (https://www.ncbi.nlm.nih.gov/bioproject/PRJNA1290883).

## Footnotes

1
https://qiime2.org/

2
https://www.arb-silva.de/

## Supplementary material

SUPPLEMENTARY TABLE S1 Water quality parameters in six freshwater exhibits over two-month period.

SUPPLEMENTARY TABLE S2 Summary of sequencing quality metrics for water samples collected from six freshwater exhibits over two-month period.

SUPPLEMENTARY FIGURE S1 Rarefaction curves of microbial communities across all samples. Rarefaction depth is represented by the number of sequences per sample on the x-axis, and rarefaction curves depict the accumulation of observed OTUs with increasing sequencing depth. Each curve corresponds to an individual sample collected in the six exhibits over the study period.

SUPPLEMENTARY FIGURE S2 Heatmap showing the temporal variation in the relative abundance of dominant bacterial genera across six exhibits over the 8-week sampling period. Columns represent samples grouped by exhibit and ordered chronologically within each exhibit (Week 1 to Week 8).
