# Supplementary figures and images for: Temporal microbiome dynamics and fish health-associated dysbiosis in freshwater aquarium systems: a case study from River Wonders Singapore
Source: Front Microbiol. 2026 May 20;17:1739391. doi: 10.3389/fmicb.2026.1739391 (PMC13230057; doi:10.3389/fmicb.2026.1739391)

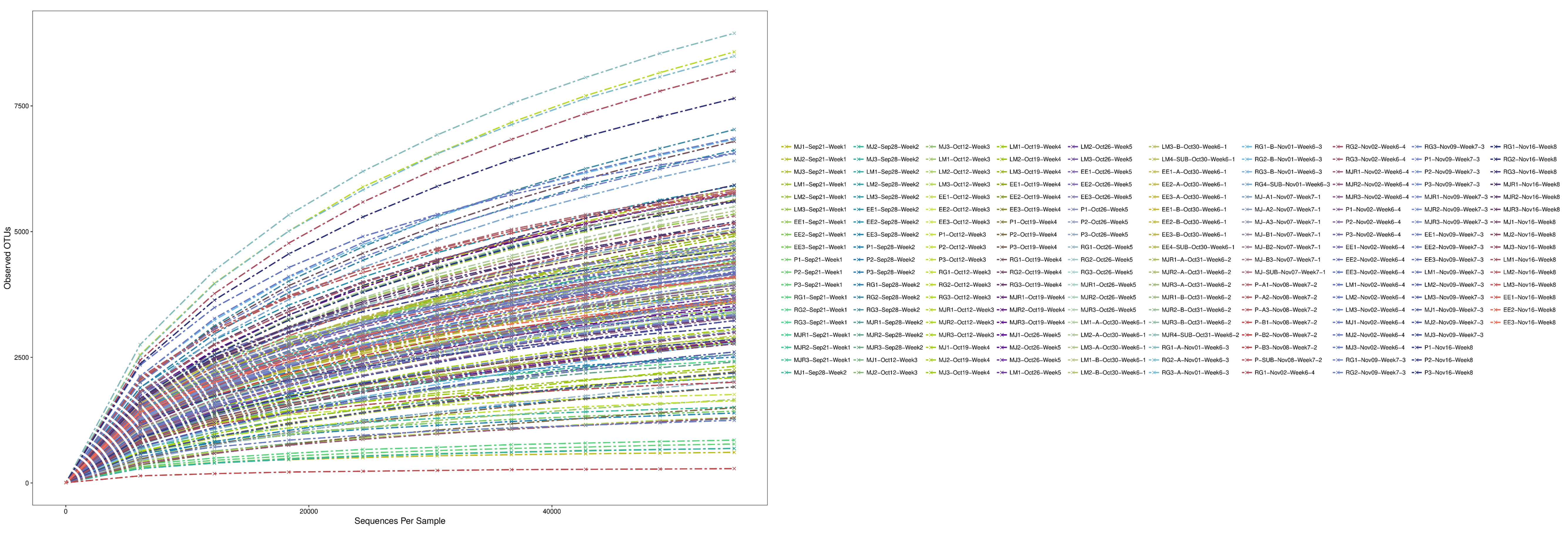

Supplement: Supplementary file 1 [file Image_1.tif]

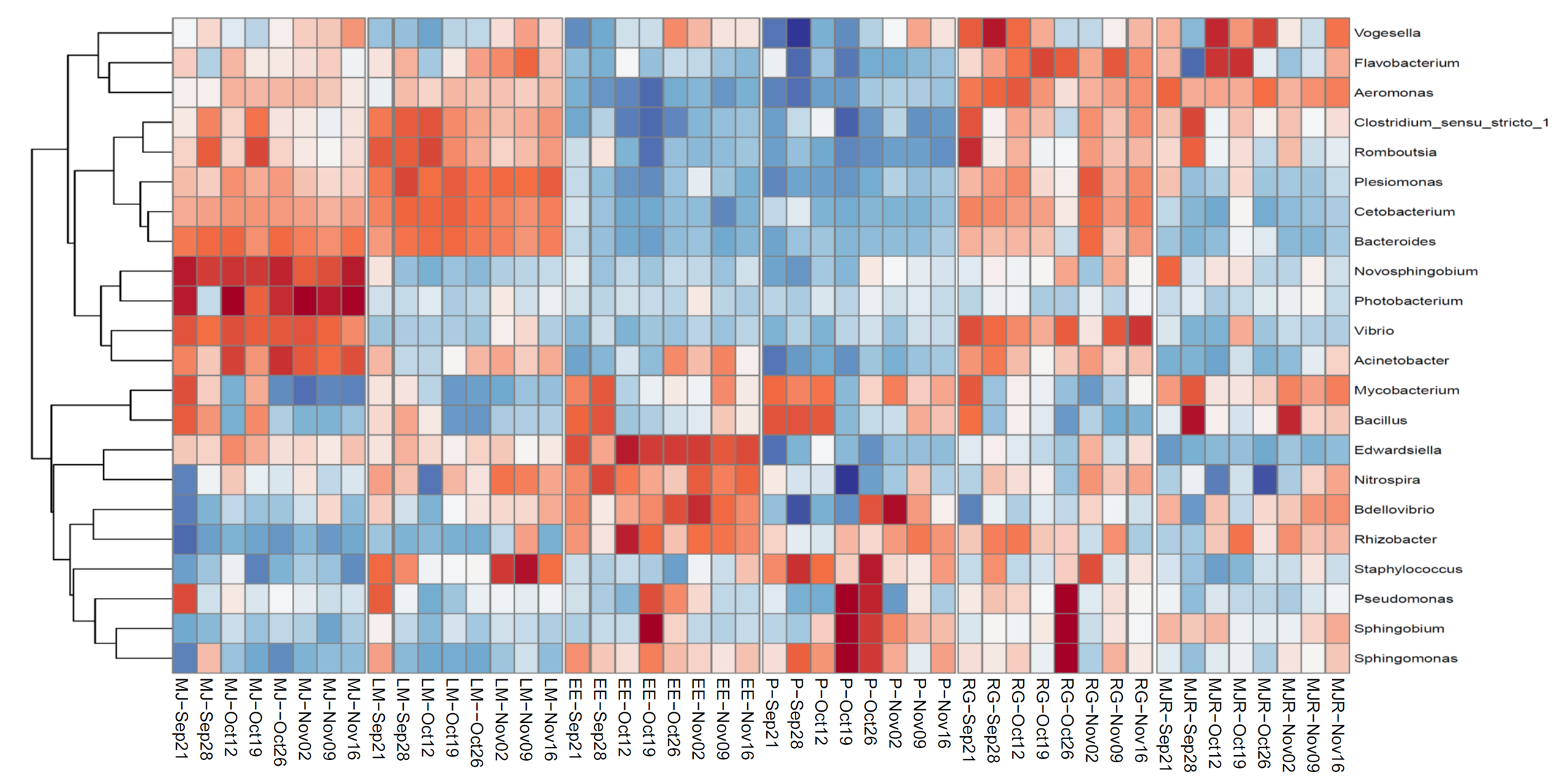

Supplement: Supplementary file 2 [file Image_2.tif]
